# Dietary supplements in integrative oncology

**DOI:** 10.3389/fonc.2026.1866767

**Published:** 2026-09-02

**Authors:** Lise Alschuler

**Affiliations:** Sonoran University of Health Sciences, Tempe, AZ, United States

**Keywords:** botanicals, dietary supplement, melatonin, mushroom, vitamin D, milk thistle

## Abstract

Dietary supplement use is highly prevalent among adults and particularly common among individuals with cancer. Despite this prevalence, oncology providers have ambivalence about endorsing dietary supplements due to important questions regarding safety, interactions, and quality assurance. In the United States and globally, the dietary supplement market has expanded substantially under a regulatory framework established by the Dietary Supplement Health and Education Act (DSHEA), yet the absence of premarket approval contributes to uncertainty and unfamiliarity among oncology providers. Constraints on treatment claims indirectly discourage large-scale clinical research, resulting in an evidence base that is dominated by epidemiologic studies, biomarker investigations, animal models, and trials conducted largely outside the conventional drug development paradigm. Within this context, integrative oncology has focused on selected agents with emerging data, including vitamin D, medicinal mushrooms, milk thistle, and melatonin. These supplements represent the most common categories and are among the most frequently used by individuals diagnosed with cancer. Collectively, these data indicate that selected dietary supplements may contribute to cancer prevention, treatment response, survival, and supportive care outcomes when integrated thoughtfully with standard therapies. This integration must be guided by rigorous quality assurance, individualized risk-benefit assessment, and ongoing research to clarify optimal adjunctive use.

## Introduction

Dietary supplement use has risen substantially over recent decades, driven by patient interest in self-directed health practices and through a growing integrative oncology provider network. Supportive care options during and after cancer treatment are increasingly commonplace. The supplement industry has experienced sustained annual growth, and the global market size and projections underscore the economic and clinical relevance of these products for oncology populations. Among individuals with cancer, dietary supplements are frequently used to address symptoms, improve quality of life, and potentially influence disease-related outcomes, often alongside conventional chemotherapy, radiotherapy, and targeted therapies. At the same time, disclosure of supplement use to oncology providers remains incomplete, creating potential safety, interaction, and quality concerns that require systematic attention in integrative oncology practice (1).

This article reviews key features of the contemporary dietary supplement landscape relevant to oncology, including regulatory context, research challenges, and third-party quality certifications, and then summarizes exemplar evidence for vitamin D, medicinal mushroom extracts, *Silybum marianum* (milk thistle), and melatonin in relation to cancer risk, treatment response, and supportive care outcomes.

## Dietary supplement use and market trends

Dietary supplements have become a major component of health-related consumer behavior, with consistent year-over-year expansion of the commercial sector. The global supplement market has grown at or above 5% to 17% per year, with a global market projected to reach $299 billion by 2030 (2). The United States dietary supplement market had over $150 billion in 2023, clearly a leading region of the global market (3). As of 2018, the U.S. market alone includes over 71,000 individual dietary supplement products (4). Population-level survey data mirror these commercial trends. Based on National Health and Nutrition Examination Survey (NHANES) data overall supplement use increased from approximately 52% in 2011–2012 to 61% in 2021-2023 (p<0.001) (5). Among adults with cancer, 66% report the use of dietary supplements (6). Despite this widespread use, only 33% of adults disclose herb or supplement use to their physicians or other conventional medical professionals and less than 51% of those with chronic conditions disclose their supplement use (7). This gap is particularly salient in oncology given the potential for interactions with chemotherapeutic agents, biologic agents and radiotherapy.

## Regulatory definition and framework

In the United States, the Dietary Supplement Health and Education Act (DSHEA) provides the foundational definition and regulatory framework for dietary supplements. DSHEA defines a dietary supplement as a product intended for ingestion that contains a “dietary ingredient” intended to supplement the diet, including vitamins and minerals, herbs and other botanicals, amino acids, dietary substances that are part of the food supply such as enzymes and live microbials (probiotics), and concentrates, metabolites, constituents, extracts, or combinations of these categories (8). The statute was conceived as landmark legislation to safeguard public safety while preserving consumer access, granting the U.S. Food and Drug Administration (FDA) authority to prohibit unsafe and mislabeled dietary supplements.

The passage of DSHEA in 1994 followed substantial public outcry against perceived governmental efforts to restrict supplement availability, and full implementation took more than a decade. Since June 2010, all U.S. dietary supplement manufacturers have been required to comply with DSHEA-related current good manufacturing practice (cGMP) requirements codified at 21 CFR Part 111. Over the same period, the dietary supplement industry in the U.S. grew from an approximately 4 billion dollar market with about 4,000 products in 1994 (9) to an estimated 150 billion dollar market in 2024 (10), encompassing over 125,000 products (11).

A central feature of the regulatory framework is that dietary supplements, unlike pharmaceuticals, have no requirement for premarket approval of safety or efficacy. Under DSHEA, supplements are considered safe until proven otherwise, whereas pharmaceuticals are considered unsafe until proven safe. Consequently, manufacturers are not required to conduct premarket clinical trials to support efficacy claims. New dietary ingredients (NDIs), however, must be supported by evidence that they are “reasonably expected to be safe,” and failure to file an NDI notification renders a product adulterated under the law. Furthermore, NDI notification is required when previously marketed ingredients are produced using substantially different methods than those in use in 1994. The Dietary Supplement and Nonprescription Drug Consumer Protection Act of 2006 adds a pharmacovigilance layer by requiring manufacturers to report serious adverse events to the FDA within 15 days of discovery and to maintain related records for at least six years (12).

## Third-party quality certification

In addition to statutory and regulatory requirements, third-party certification programs have emerged as an important mechanism for assessing dietary supplement quality. The NSF International Dietary Supplements Certification Program is a prominent example of an independent, nonprofit initiative that evaluates manufacturing practices and product composition (13). Core components of this program include twice-yearly on-site audits, verification that supplement contents match the label, assurance that all ingredients are fully disclosed on the label, and assessment that products do not contain unacceptable levels of contaminants. Manufacturers use such certification to demonstrate cGMP compliance and to differentiate their products on the basis of quality practices.

Notwithstanding these strengths, NSF certification is not synonymous with continuous or comprehensive quality verification because not every product lot or raw ingredient is necessarily tested. The program certifies compliance with DSHEA-related requirements rather than establishing new regulatory standards. Other programs, such as the U.S. Pharmacopeia (USP) dietary supplement verification program, provide additional avenues for quality assessment. However, USP verification is monograph-based quality standards and most dietary supplement ingredients do not have USP monographs (14). ConsumerLab.com is a for-profit, subscription-based testing service that independently tests products for potency and heavy metal contamination as a marker for purity (15). Products which pass these tests can carry a “CL Approved Quality Product” seal. For integrative oncology practice, awareness of third-party certification can help clinicians guide patients toward products that meet recognized quality benchmarks, while still emphasizing that certification does not guarantee clinical efficacy.

## Research incentives and constraints

The existing regulatory structure creates disincentives for robust clinical research on dietary supplements, including those used in oncology. Because DSHEA restricts treatment claims for supplements and does not tie regulatory approval to the demonstration of efficacy, there is limited economic incentive for manufacturers to sponsor large, high-quality randomized controlled trials. The relatively low unit price of many supplements further constrains potential returns on research investment for commercial entities.

In the U.S. context, clinical studies are typically defined as research in individuals with existing illness or at risk for disease, which can increase complexity and cost when designing trials aimed at cancer prevention or adjunctive treatment outcomes. As a result, much of the evidence base for dietary supplements consists of epidemiological correlation studies, biomarker-focused investigations, animal models, and prospective trials conducted outside the United States or by independent academic centers rather than by industry sponsors. In integrative oncology, these constraints translate into significantly heterogeneous literature with varying quality and generalizability. This underscores the importance of careful appraisal and contextualization of available evidence for each agent.

## Vitamin D: epidemiological and clinical data in oncology

Vitamin D has been extensively investigated in relation to cancer risk and outcomes, with particular emphasis on breast, colorectal, prostate, and lung cancers. A pooled analysis of two randomized controlled trials (N = 1129 and N = 2196) and a prospective cohort (N = 1713) evaluated serum 25-hydroxyvitamin D [25(OH)D] concentrations and breast cancer incidence over a median four-year follow-up (16). In this analysis, women with serum 25(OH)D levels at or above 60 ng/mL (150 nmol/L) had an 82% lower incidence of breast cancer compared with those with levels below 20 ng/mL (50 nmol/L), suggesting a strong inverse association between vitamin D status and breast cancer risk.

A dose-response meta-analysis of six cohort studies conducted between 1974 and 2017 (N = 5984) further examined circulating 25(OH)D levels and overall survival among women with breast cancer (17). A pooled hazard ratio comparing the highest versus lowest 25(OH)D categories indicated improved survival in those with higher vitamin D status, and a linear dose-response relationship was identified above a threshold of 23.3 nmol/L (9.3 ng/mL). Specifically, in this range, each 4 nmol/L (1.6 ng/mL) increase in circulating 25(OH)D corresponded to a 6% decrease in overall mortality, an 8 nmol/L (3.2 ng/mL) increase to a 12% decrease, and a 10 nmol/L (4 ng/mL) increase to a 14% decrease in overall mortality. These findings support a highly significant linear association between higher circulating 25(OH)D levels and improved overall survival in breast cancer patients.

A separate meta-analysis of prospective cohort studies among breast cancer survivors (N = 1666, with 30 studies included) evaluated both breast cancer–specific and overall mortality in relation to vitamin D status (18). In this analysis, high blood levels of 25(OH)D were associated with a 42% reduction in breast cancer mortality (pooled relative risk 0.58, 95% CI 0.40–0.85) and a 49% reduction in overall mortality (pooled relative risk 0.61, 95% CI 0.48–0.79), with no evidence of substantial heterogeneity or publication bias.

Vitamin D has also been examined in relation to prostate cancer. A nested case–control study within the Prostate, Lung, Colorectal, and Ovarian (PLCO) Cancer Screening Trial included 749 patients with incident prostate cancer diagnosed one to eight years after baseline blood draw and 781 matched controls (19). Overall, no statistically significant trend in total prostate cancer risk was observed across season-standardized serum 25(OH)D quintiles. However, men in the lowest quintile had an increased risk of aggressive disease, defined by Gleason score ≥ 7 or clinical stage III/IV, in models adjusted for matching factors, study center, and history of diabetes. Mechanistic considerations support a potential anti-cancer role for vitamin D, including the ability of many cell types to convert circulating 25(OH)D to the active metabolite 1,25-dihydroxyvitamin D [1,25(OH)D], whereas prostate cancer cells often lose this hydroxylation capacity and may therefore rely on circulating 1,25(OH)D (20). This could explain why high calcium intake or milk intake is associated with increased risk of advanced prostate cancer since serum calcium suppresses circulating 1,25(OH)D (21), thereby further limiting the antiproliferative and differentiation benefits of active vitamin D in prostate cancer cells.

Further complexity is highlighted by analyses from the Selenium and Vitamin E Cancer Prevention Trial, which included 1,731 cases and 3,203 cohort participants (22). In this context, U-shaped associations were observed between vitamin D status and total prostate cancer risk, such that both low and high 25(OH)D concentrations were associated with increased risk, particularly for high-grade disease. Vitamin D may also exert stage-specific effects; in men with Gleason 8–10 prostate cancer, higher 25(OH)D levels were associated with decreased risk, especially among those older than 65 years, whereas in men with Gleason 2 disease, higher 25(OH)D was associated with a modest increase in risk.

In lung cancer, a cohort of 447 patients with early-stage non–small-cell lung cancer (NSCLC) was followed for a median of 72 months (range 0.2–141), during which 161 recurrences and 234 deaths occurred (23). Comparing the highest versus lowest quartiles of baseline serum 25(OH)D, the adjusted hazard ratio for overall survival was 0.74 (95% CI 0.50–1.10; P for trend = .07), with a stronger association observed in patients with stage IB–IIB disease (adjusted hazard ratio 0.45, 95% CI 0.24–0.82; P for trend = .002) but not in those with stage IA disease (adjusted hazard ratio 1.10, 95% CI 0.62–1.96; P for trend = 0.53). Similar patterns were seen for recurrence-free survival, suggesting that vitamin D status may influence outcomes in specific subgroups of early-stage NSCLC.

Taken together, these data suggest that vitamin D may contribute to cancer prevention and improved survival after diagnosis in selected contexts, while also indicating that the relationship between vitamin D status and cancer risk can be non-linear and stage-dependent, particularly for prostate cancer. It is important to note that the overall literature on vitamin D and general health outcomes remains heterogeneous, with notable gaps. However, existing reviews suggest that a daily intake of 2,000 IU (50 mcg) is generally sufficient to raise and maintain serum 25(OH)D levels above 50 nmol/L (20 ng/mL) in approximately 99% of the population and above 75 nmol/L (30 ng/mL) in about 90% of adults. This intake level has not been associated with evidence of harm, including among individuals with adequate baseline vitamin D status (24). Nonetheless, medical supervision of vitamin D status and supplementation is advisable, particularly in those with metabolic and renal disease.

## Medicinal mushrooms in integrative oncology

Medicinal mushrooms represent a diverse group of fungi with immunomodulatory properties relevant to oncology, mediated largely through beta-glucan polysaccharides and other bioactive constituents. All mushrooms contain protein-bound beta-glucans within their cell walls, present primarily in the fruiting body but also in the mycelium (25). The typical polysaccharide backbone consists of beta-(1,3)-linked D-glucose units, with additional beta-D-glucose branches attached via 1,6 linkages; 1,3 beta-glucans are characteristic of fungi and yeast. Mushroom-derived 1,3 beta-glucan chains are arranged as triple helices, which are considered more immunologically potency than single-helix beta-glucans (26).

Beyond beta-glucans, fruiting bodies contain terpenoids (such as hericenones), lectins, sterols, adenosine compounds, carbohydrates, and fats, while mycelia provide additional secondary metabolites including di- and triterpenes (such as erinacines), vitamins, minerals, and ergosterol. Beta-glucans are fermented by intestinal microbiota into bioactive short-chain fatty acids (SCFAs) such as acetate, propionate, and butyrate (27). The SCFAs exert anti-inflammatory effects by modifying inflammatory cytokine production in antigen presenting cells and induce Th1 cell differentiation (28).

Beta-glucans can interact directly with immune cells or be internalized by intestinal M cells and transported to gut-associated lymphoid tissue (GALT) Peyer’s patches (29). Within Peyer’s patches and mesenteric lymph nodes, dendritic cells process mushroom polysaccharides and present them to T-lymphocytes, thereby influencing downstream immune responses. Dendritic cells are among the most potent antigen-presenting cells for activating naïve CD4- and CD8- T lymphocytes.

At the cellular level, mushroom polysaccharides are recognized by pattern-recognition receptors, including Dectin-1 (a C-type lectin receptor) with Toll-like co-receptors (TLRs), that detect pathogen-associated molecular patterns (PAMPs) (30). Because beta-glucans are large molecules that do not readily penetrate cells, their initial immunomodulatory effect occurs through binding to pattern recognition receptors on macrophages, dendritic cells, and natural killer (NK) cells. In this context, beta-glucans act as fungal PAMPs for the innate immune system. β-1,3/1,6-glucans stimulate a predominantly T helper 1 (Th1) response characterized by increased production of cytokines such as interleukin-2 (IL-2), interleukin-12 (IL-12), and interferon-gamma (IFN-γ), while Th2 (humoral) responses are generally suppressed or less prominently activated (31).

Dendritic cells in GALT and macrophages engulf beta-glucans via TLR2/6 receptors and migrate to the spleen, lymph nodes, and bone marrow (32). In the bone marrow, macrophages degrade beta-glucans into smaller soluble beta-1,3-glucan fragments. These fragments are then released and taken up by circulating innate immune cells—neutrophils, NK cells, monocytes, and dendritic cells—through complement receptor 3 (CR3). CR3 engagement primes these cells to phagocytose targets that express iC3b, including opsonized tumor cells and infected cells, and also enhances the expression of anti-inflammatory cytokines such as transforming growth factor-beta 2 (TGF-β2) and interleukin-10 (IL-10), helping modulate the inflammatory response. Furthermore, mycelium and fruiting-body extracts from multiple mushroom species have been shown to activate innate immunity and increase interferon production while simultaneously exerting anti-inflammatory effects, partly through increased production of interleukin-1 receptor antagonist (IL-1Ra), which counteracts the pro-inflammatory actions of interleukin-1 beta (IL-1β) (33).

## Reishi (*Ganoderma lucidum*) in cancer treatment

Reishi mushroom has a long history of use in traditional medicine and has been evaluated as an adjunctive therapy in cancer. A Cochrane review identified five randomized controlled trials in which patients with various cancers received *Ganoderma lucidum* at a daily dose of 3 grams (34). This review concluded that *G. lucidum* was well-tolerated and that CD3, CD4 and CD8 cells increased with use. This meta-analysis showed that patients who received G. lucidum with chemotherapy or radiotherapy experienced positive tumor responses compared with those receiving conventional therapy alone (relative risk 1.50; 95% confidence interval 0.90–2.51; p = 0.02), whereas G. lucidum monotherapy did not achieve comparable regression rates. This review is substantially limited by small (five randomized trials with a total of 373 participants), methodologically weak trials (some trials lacked clear descriptions of randomization and blinding and there was heterogeneity in products and dosing schedules) with short follow-up (most studies were only 1–3 months).These Cochrane reviews indicate that it not clear if *G. lucidum* prolongs long-term survival, but that it may be considered as an adjunct to conventional treatment due to its potential to enhance tumor response and stimulate host immunity. Notably, there are no clinical studies which demonstrate harm from reishi mushroom.

### Turkey tail (*Coriolus versicolor*) and solid tumors

Turkey Tail mushroom, particularly its polysaccharide K (PSK) and polysaccharopeptide (PSP) fractions, has been studied extensively as an adjunctive therapy in colorectal and other solid tumors. A Cochrane systematic review published in 2022 included seven randomized controlled trials of patients with colorectal cancer receiving chemotherapy or radiotherapy with PSK interventions lasting from four weeks to three years (35). Low-certainty evidence indicated an improvement in five-year overall survival with PSK (relative risk 1.08, 95% CI 1.01–1.15), corresponding to a number needed to treat (NNT) of 16 (95% CI 9–70). The certainty of evidence was downgraded because of heterogeneity in trial design, including variability in PSK preparations (raw, decoction, capsule, tablet, tincture, extract, injection) fungal parts used (cap, stem, mycelium, whole fungus), and dosing regimens.

Beyond colorectal cancer, a systematic review and meta-analysis of 13 randomized controlled trials, including 2,587 patients with stage I–IV solid tumors, compared standard- of-care conventional treatment with or without the addition of 3 grams daily of Turkey Tail preparations, including isolated PSK or PSP extracts (36). The addition of Turkey Tail resulted in a 9% absolute reduction in five-year mortality, corresponding to an NNT of 11 (p < 0.00001), and the intervention was generally well tolerated. This applicability of meta-analysis is limited by the inclusion of differing cancer types, stages, treatment regimens as well as the fact that the chemotherapy regimens are not always consistent with regimens used currently. Thus, extrapolation of these study findings to contemporary drug regimens is speculative. These findings signal the potential role of Turkey Tail polysaccharides as adjuncts to conventional cancer therapy, although variability in study populations, tumor types, and formulations in addition to heterogenous and older trials underscores the need for further high-quality prospective trials. Again, there are no studies demonstrating harm from turkey tail mushroom.

## Milk thistle (*Silybum marianum*) in oncology-related hepatic and treatment support

*Silybum marianum*, commonly known as milk thistle or St. Mary’s thistle, has a long tradition of use for liver protection and hepatotoxicity mitigation, and its constituents have attracted interest in oncology for managing treatment-related hepatic and other toxicities. Milk thistle is a member of the Asteraceae (daisy) family, and the seeds (fruits) contain 1.5% to 3% flavolignans collectively known as silymarin, as well as oils (including oleic and palmitic acids), sterols (cholesterol, campesterol, stigmasterol, sitosterol), and mucilage. Silymarin is a complex mixture including silybin (approximately 50% - 70%), silydianin, and silychristin, with silybin considered the most biologically active flavonolignan (37). Structurally, these compounds are part flavonoid (e.g., taxifolin) and part lignan, linked via oxidative coupling. Extracted silymarin typically contains 70%–80% silymarin flavonoids and 20%–35% fatty acids.

Mechanistically, milk thistle exerts multiple hepatoprotective actions, including decreased intracellular production and release of hepatic transaminases (AST, ALT, GGT, alkaline phosphatase), improved hepatocellular membrane integrity, and upregulation of the Nrf2- mediated antioxidant response while downregulating NF-κB signaling (38). Silymarin increases enzymatic antioxidants such as glutathione and selenium transferase, and reduces pro-inflammatory cytokines by impeding NLRP3 inflammasome activation and the release of tumor necrosis factor-alpha (TNF-α), interleukin-6 (IL-6), interleukin-1 beta (IL-1β), interleukin-12B, and transforming growth factor-beta (TGF-β), among others (39). It has both pro-apoptotic and anti-apoptotic effects, mitigating oxidative stress–induced activation of caspases and modulating inflammatory signaling. Additional actions include reduced hepatic fibrosis via decreased activation of hepatic stellate and Kupffer cells, reduced deposition of extracellular matrix proteins, decreased collagen and procollagen production, and increased matrix metalloproteinase-2 (MMP-2), collectively limiting fibrotic progression. Silymarin also blocks and inactivates oxidative hepatotoxins and promotes hepatic regeneration by enhancing RNA and RNA polymerase I synthesis, thereby supporting repair of damaged hepatocytes.

Clinical studies in oncology and related settings suggest potential benefits in preventing or mitigating treatment-related hepatic injury and other toxicities. In a randomized, double- blind, placebo-controlled pilot trial of 50 children with acute lymphoblastic leukemia and chemotherapy-associated hepatic toxicity, participants received either standardized milk thistle extract at 5 mg/kg or placebo (40). No significant differences were observed in the frequency of side effects, incidence and severity of toxicities, or infections between groups; however, at day 56, the milk thistle group had a significantly lower AST level (p = .05) and a trend toward lower ALT (p = .07), suggesting a potential hepatoprotective effect. This trial is limited by the small sample size and short duration. Furthermore the lack of differences in severity of chemotherapy toxicities or other clinical outcomes limits the clinical utility of the biochemical findings. The authors also note that the dose of milk thistle used may be insufficient, thereby weakening the certainty of the dose-response.

In a randomized, triple-blind, placebo-controlled trial of 30 women with non-metastatic breast cancer and at least grade 2 liver injury by ultrasound, participants were enrolled during the fourth and final cycle of dose-dense doxorubicin/cyclophosphamide (AC-T) prior to paclitaxel (41). Over one month, they received either 140 mg of silymarin three times daily or a control milk thistle preparation without silymarin. After one month, there was a non significant trend toward reduced fatty liver in the silymarin group, but no significant differences in liver stiffness or in liver function tests. This study is clearly limited by the small sample size and given the study duration, only addresses acute AC-T chemotherapy-induced toxicity.

A pilot study of 70 patients with metastatic colorectal cancer receiving first-line FOLFIRI (5-fluorouracil, leucovorin, irinotecan) plus bevacizumab evaluated oral milk thistle extract at 150 mg three times daily for one week (42). Compared to controls, the milk thistle group experienced less diarrhea (5.7% vs 14.6%; p = 0.002) and less nausea (27% vs 40.2%; p = 0.005), while differences in liver toxicity were not observed, potentially due to the short intervention duration. This study was not blinded, therefore the placebo-effect cannot be ruled out, and the small single-site sample limit generalizability. In a double-blind, randomized, placebo-controlled pilot trial of 27 patients with head and neck cancer receiving radiotherapy plus cisplatin every 21 days for three cycles, silymarin 420 mg daily in three divided doses was administered from the first day of radiotherapy through the six-week course (43). Although mucositis scores increased in both groups, onset was delayed and severity was significantly lower in the silymarin group (p < 0.05).

Another randomized, double-blind, placebo-controlled trial of 40 patients with gastrointestinal cancers receiving capecitabine examined topical silymarin gel 1% applied to the palms and soles twice daily for nine weeks, beginning with chemotherapy (44). Median hand-foot syndrome scores were significantly lower in the silymarin group at nine weeks (p < 0.05), and although scores increased over time in both groups, silymarin recipients developed hand-foot syndrome later and with less severity. Both of these studies are limited by their small and single site patient samples, thereby weakening generalizability and the studies did not include clinical functional outcomes such as ability to eat or dose modification thereby making the clinical significance uncertain.

Silymarin has poor aqueous solubility and correspondingly low bioavailability, which can be improved through formulation as nanoparticles or phytosomes (phosphatidylcholine complexes) (45). Clinical experience and published data suggest that milk thistle is generally well tolerated and considered safe during pregnancy and breastfeeding. *In vitro* and *in vivo* studies indicate that silymarin may inhibit drug efflux pumps, potentially reducing resistance to antibiotics and oral chemotherapeutic agents, and thus may be well suited for use alongside hepatotoxic antibiotics.

With respect to drug–herb interactions, *in vivo* studies have shown inhibition of cytochrome P450 enzymes cyp3A4 and cyp2C9 by silymarin flavonoids, but these effects have not been consistently demonstrated in humans and are not considered clinically significant in most settings (46). One study reported that silymarin 140 mg three times daily for 14 days increased the area under the curve (AUC) of losartan in individuals with the CYP2C9*1/*1 genotype, indicating that specific pharmacokinetic interactions may occur in susceptible subgroups (47). These findings reinforce the importance of individualized assessment when integrating milk thistle into oncology care.

## Melatonin as an adjunctive oncology agent

Melatonin, a pineal hormone with circadian regulatory and antioxidant properties, has been extensively studied in oncology as an adjunct to chemotherapy and radiotherapy. Despite the antioxidant actions of melatonin, preclinical and clinical studies have failed to find any interference with the efficacy of chemotherapy and radiation. Preclinical studies demonstrate an antioxidant effect in healthy cells with a conditionally pro-oxidant effect in malignant cells (48). This differential effect with overall oncostatic effects has been demonstrated in a number of clinical trials. A meta-analysis of eight randomized controlled trials in patients with solid tumors (total n = 761) evaluated oral melatonin at 20 mg nightly in combination with chemotherapy or radiotherapy (49). Compared with control groups receiving conventional therapy alone, melatonin significantly improved complete and partial remission rates (16.5% vs 32.6%; relative risk 1.95, 95% CI 1.49–2.54; p < 0.00001) and increased one-year survival (28.4% vs 52.2%; relative risk 1.90, 95% CI 1.28–2.83; p = 0.001). Melatonin co-administration also reduced chemotherapy- and radiotherapy-related side effects, including thrombocytopenia (19.7% vs 2.2%; relative risk 0.13, 95% CI 0.06–0.28; p < 0.00001), neurotoxicity (15.2% vs 2.5%; relative risk 0.19, 95% CI 0.09–0.40; p < 0.0001), and fatigue (49.1% vs 17.2%; relative risk 0.37, 95% CI 0.28–0.48; p < 0.00001), with consistent effects across different tumor types and no severe adverse events reported. The strength of this meta-analysis is weakened by the relatively small number of trials (8) and total participants (761), clinical heterogeneity (cancer type, stage and treatment regimens), and possible bias in the heavy reliance on published positive studies, thus this evidence should be considered as suggestive only.

More detailed examples illustrate these findings in specific cancer populations. In a study of 30 patients with metastatic colorectal cancer who had disease progression after at least one prior 5-fluorouracil–containing chemotherapy regimen, participants were randomized to receive irinotecan (CPT-11) alone or irinotecan plus melatonin 20 mg daily administered during the dark period (50). The proportion of patients achieving disease control (complete response, partial response, or stable disease) was significantly higher in the melatonin plus chemotherapy group than in the chemotherapy-only group (12 of 14 vs 7 of 16; p < 0.05). The small sample size and single-site enrollment limit the statistical power of this study and these findings may not generalize to broader populations of patients with metastatic colorectal cancer. Also of note is that this study utilizes an older chemotherapy regimen which is not reflective of multi-agent treatments, calling into question its clinical relevance without further studies.

In another trial, 100 patients with previously untreated metastatic NSCLC were followed for 72 months (51). One group received chemotherapy alone with cisplatin 20 mg/kg day and etoposide 100 mg/day for three consecutive days every 21 days, while the other group received the same chemotherapy plus melatonin 20 mg daily, initiated seven days before chemotherapy and continued throughout treatment. The melatonin group had higher rates of objective clinical response (complete plus partial response), improved disease stabilization, and lower rates of progressive disease, along with reduced incidence of neurotoxicity and thrombocytopenia, with statistically significant differences in these toxicities (p < 0.01). This study also utilized a dated chemotherapy regimen, limiting its clinical relevance now. Furthermore, the study did not use a placebo and the methodology does not adequately describe the procedures for randomization, thereby raising concerns about selection and assessment bias.

Collectively, these data support the potential of melatonin to improve progression-free survival, overall survival, and quality of life while mitigating treatment-related adverse effects when used adjunctively with conventional oncology regimens. Clearly, more robust and larger clinical studies with modern chemotherapy regimens are needed to better elucidate the effect of melatonin on quality and quantity of life of individuals diagnosed with cancer.

Melatonin is considered to have low and highly variable bioavailability due to hepatic first-pass, with approximately 15% of an oral dose measured in the serum approximately 50 minutes after dosing (52). Melatonin is well-tolerated. In a systematic review of 50 controlled studies, half reported no significant adverse events, while the remaining studies reported minor, short-lived symptoms from melatonin such as fatigue and mood changes (53). Importantly, higher doses (above 10mg daily) do not result in any increases in serious adverse events by incidence or severity (54). Melatonin is a substrate for CYP1A2 and its bioavailability is thus enhanced by CYP1A2 inhibitors such as fluvoxamine and caffeine (55).

## Clinical implications for integrative oncology practice

Given that approximately almost 2 out of every 3 patients is using dietary supplements regularly, integrative oncology clinicians must systematically assess and document supplement use as part of routine care. Inquiry should encompass product names, doses, duration, and manufacturer information, as well as whether products carry third-party quality certifications. This information is critical to evaluating potential benefits, synergistic effects with conventional treatment, and risks of pharmacokinetic or pharmacodynamic interactions, particularly in settings involving narrow therapeutic index agents or complex polypharmacy.

The evidence synthesized here suggests that vitamin D may contribute to cancer prevention and improved survival in selected populations, that mushroom extracts, especially *Ganoderma lucidum* and *Coriolus versicolor*, may modestly enhance response rates and survival when added to standard therapies for a variety of solid tumors. *Silybum marianum* (Milk thistle) emerges as a candidate for hepatic and mucosal protection and for reducing specific chemotherapy-related toxicities, though limited by small and heterogeneous studies. Melatonin can improve treatment response, survival, and tolerability in patients receiving chemotherapy and radiotherapy. Across all of these dietary supplement examples, the substantial but heterogeneous evidence base underscores the importance of ongoing research, critical appraisal, and shared decision-making grounded in patient values and goals.
